# Small Molecule Therapeutics in the Pipeline Targeting for Triple-Negative Breast Cancer: Origin, Challenges, Opportunities, and Mechanisms of Action

**DOI:** 10.3390/ijms25116285

**Published:** 2024-06-06

**Authors:** Nneoma James, Esther Owusu, Gildardo Rivera, Debasish Bandyopadhyay

**Affiliations:** 1 School of Integrative Biological and Chemical Sciences, The University of Texas Rio Grande Valley, 1201 West University Drive, Edinburg, TX 78539, USA; nneoma.james01@utrgv.edu (N.J.); esther.owusu01@utrgv.edu (E.O.); 2 Laboratorio de Biotecnología Farmacéutica, Centro de Biotecnología Genómica, Instituto Politécnico Nacional, Reynosa 88710, Mexico; giriveras@ipn.mx; 3 School of Earth Environment & Marine Sciences (SEEMS), The University of Texas Rio Grande Valley, 1201 West University Drive, Edinburg, TX 78539, USA

**Keywords:** triple-negative breast cancer, breast cancer, estrogen receptor, human epidermal growth factor receptor 2, progesterone receptor, small molecules, chemotherapy, chemoresistance, immunotherapy, signaling pathways

## Abstract

Triple-negative breast cancer (TNBC) cells are devoid of estrogen receptors (ERs), progesterone receptor (PRs), and human epidermal growth factor receptor 2 (HER2), and it (TNBC) counts for about 10–15% of all breast cancers. TNBC is highly invasive, having a faster growth rate and a higher risk of metastasis and recurrence. Still, chemotherapy is one of the widely used options for treating TNBC. This study reviewed the histological and molecular characterization of TNBC subtypes, signaling pathways that are aberrantly expressed, and small molecules targeting these pathways, as either single agents or in combination with other therapeutic agents like chemotherapeutics, immunotherapeutics, and antibody–drug conjugates; their mechanisms of action, challenges, and future perspectives were also reviewed. A detailed analytical review was carried out using the literature collected from the SciFinder, PubMed, ScienceDirect, Google Scholar, ACS, Springer, and Wiley databases. Several small molecule inhibitors were found to be therapeutics for treating TNBC. The mechanism of action and the different signaling pathways through which the small molecules exert their effects were studied, including clinical trials, if reported. These small molecule inhibitors include buparlisib, everolimus, vandetanib, apatinib, olaparib, salidroside, etc. Some of the signaling pathways involved in TNBC, including the VEGF, PARP, STAT3, MAPK, EGFR, P13K, and SRC pathways, were discussed. Due to the absence of these biomarkers, drug development for treating TNBC is challenging, with chemotherapy being the main therapeutic agent. However, chemotherapy is associated with chemoresistance and a high toxicity to healthy cells as side effects. Hence, there is a continuous demand for small-molecule inhibitors that specifically target several signaling pathways that are abnormally expressed in TNBC. We attempted to include all the recent developments in this field. Any omission is truly unintentional.

## 1. Introduction

Cancer is the USA’s second leading cause of death after heart disease, posing a threat to global health [1]. Though chemotherapy is an effective therapy, some cancer cells resist chemotherapy by changing their mechanism of action, making them less susceptible to chemotherapeutic agents [1]. However, there are more than 200 types of cancer possible in humans; breast cancer (BC) has been reported to be the second most diagnosed cause of cancer death in the USA, as advanced and metastatic breast cancer is hard to treat [2]. BC is one of the leading causes of mortality from cancer in women [3], which has reduced with the advancement in technology and novel diagnostic and treatment procedures [4]. In 2020, about 279,100 new cases and 42,000 deaths due to BC were recorded in the USA [1], and about 1 in 8 women might develop invasive breast cancer in the US alone [5]. From data published by the American Cancer Society, 300,000 new cases of invasive BC and 56,000 cases of ductal carcinoma in situ (DCIS) were recorded in the USA in 2023. In 2030, about 2.3 million new cases are likely to occur, with more than 44,000 deaths [6].

BC is observed more often among Hispanic [7] and Black-American women with a poor survival rate, compared to Asians and Europeans, who have a better survival rate with less incidence of BC [1]. BC can be grouped into different subtypes based on the presence of molecular biomarkers such as estrogen receptors (ERs), human epidermal growth factor receptor 2 (HER2), and progesterone receptors (PRs) [8]. The subtypes of BC are Luminal A (ER-positive, HR-positive, and HER2-negative), luminal B (ER-positive, PR-negative, and HER2-positive), *HER2* overexpressing, normal-like breast cancer, and basal-like/triple-negative breast cancer (ER-negative, HER2-negative, and PR-negative). Due to the absence of ERs, PRs, and HER2 receptors, the basal-like breast cancer (BLBC) subtype shows similarity to triple-negative breast cancer (TNBC) with some differences in their gene expression, as only 77% of BLBCs were TNBC, while 71% of TNBCs were BLBCs [8,9]. TNBC is so called because it presents as negative for ERs, PRs, and HER-2 expressions, making it the subtype of BC with the worst prognosis because of its strong metastatic, invasive nature and its high recurrence [10,11]. The absence of these biomarkers is a great challenge in treating TNBC, and its survival rate is low, while its metastasis is high compared to other types of BC. TNBC accounts for 15–20% of recently diagnosed breast carcinoma [12]. The occurrence of TNBC is more frequent in younger women, as an analysis of 117 TNBC patients reported that 63% were women under 50 years old [8]. Many treatment strategies, including chemotherapy, immunotherapy, and targeted therapy, have been employed to treat TNBC. However, based on their ability to work on targeted therapeutic sites, small-molecule targeted drugs are promising in cancer therapeutics as they help to reduce toxicity to healthy cells, while enhancing precision medicine.

Different therapeutics are available for cancer treatments based on their subtype, including chemotherapy, immunotherapy, surgery, hormone therapy, and radiotherapy [13]. Chemotherapy is the mainstay treatment for TNBC, but it has a short-lived response with a median overall survival (OS) of 12 to 18 months, which is lower than that of other subtypes [14]. The drawbacks of chemotherapy include chemoresistance, an increase in cancer stem cells that are capable of renewal, and toxicity to healthy human cells due to its non-specific targeting. [1,15,16]. Nanoparticle drug delivery systems can potentially increase the efficacy of this form of cancer treatment. There are various advantages of nanoparticle drug delivery systems over standard medication administration methods. They can, for example, be tailored to mainly target cancer cells, while causing minimal harm to healthy cells. They can also increase drug bioavailability, meaning more medicine reaches its intended target. Furthermore, nanoparticle drug delivery methods can aid in resolving some of the issues associated with conventional chemotherapy, such as drug resistance and toxicity [10]. For the past two decades, a shift has been made from broader cytotoxic drugs to targeted drugs that are specific to cancer cells, thereby protecting healthy cells, leading to a high potency and less toxicity. Targeted drugs are divided into small molecules and macromolecules, and the first small-molecule drug (tyrosine kinase inhibitor) was approved in 2001 by the US Food and Drug Administration (FDA). Some factors, including cost, patient acceptance, and pharmacokinetics, have significantly increased the advantage of these small molecules over macromolecules (e.g., antibody–drug conjugates, monoclonal antibodies, polypeptides, etc.) [17].

Small molecule therapeutics (natural, synthetic, and semi-synthetic) are an emerging and widely explored area in drug development research. In 400 BC, Hippocrates (the father of medicine) historically recommended the water extract of willow bark as a painkiller, in which the active ingredient (principle) was salicin; this undergoes metabolism in the human body to generate saligenin, which is further metabolized in the liver to give salicylic acid, a small molecule having anti-inflammatory and antipyretic activities. Although small molecule therapeutics are well known, the first definition of small molecules from a medicinal perspective was introduced by Christopher A. Lipinski in his famous RO5 [18,19,20], based on the fact that most orally administered drugs are small organic molecules. Insights are being gained in relation to the use of small organic molecules in the development of novel therapeutics, due to their good pharmacokinetic properties, patients’ acceptance, cost, and storage. Most of the drugs approved by the U.S. FDA are small organic molecules. According to the RO5, small-molecule drugs should have a molecular weight of less than 500 Da; fewer than 10 hydrogen bond acceptors (total number of N and O atoms); fewer than 5 hydrogen bond donors (total number of N–H and O–H bonds); and the calculated LogP (CLogP) should be less than 5, where the letter “P” indicates partition coefficient in *n*-octanol-water (ClogP < 5). The ability of small-molecule drugs to cross the blood–brain barrier to penetrate and reach intracellular targets adds to their potency as cancer therapeutics in targeting gene mutations and aberrant signaling pathways [21]. However, in the subsequent years, a few more parameters have been added to the RO5 to predict the drug-likeness of small molecules. These include molar refractivity, polar surface area, rotatable bonds, etc. Some commonly used small-molecule drugs are aspirin, atenolol, diazepam, diclofenac, ibuprofen, felodipine, omeprazole, phenytoin, and many others [22,23,24,25]. This review will shed light on the molecular characterization of TNBC, single drug targets, drug combinations, and different small molecules that are targeted to cause regulated or programmed cell death for the treatment of TNBC.

## 2. Histological and Molecular Characterization of Triple-Negative Breast Cancer

Of all the BC subtypes, TNBC is the most aggressive and heterogeneous [5]. TNBC comprises roughly 10–15% of all diagnosed breast cancer cases [26]. TNBC is highly heterogeneous in terms of stromal and immunological microenvironment, genomic integrity, oncogenic signatures, and metabolism processes [27,28,29]. TNBC is a higher staged nuclear-grade cancer with a very low prognosis and intense mitotic activity [30]. The identification of the different subtypes and molecular hallmarks of TNBC is of vital importance in the development of therapeutic approaches [5,31]. TNBC can be classified in two ways—histological classification and molecular classification. TNBC can also be classified into HRD (homologous recombination deficiency) and HRP (homologous recombination proficiency) subtypes. The definition of HRD or HRP should be broader than that of BRCA mutation or wild type [28]. We have focused on the description of BRCA status, not HR (homologous recombination) status, for the sensitivity of PARP inhibitors. This can be expanded to HR-related scope to include more patients who may benefit from the therapies.

### 2.1. Histological Classification

Generally, the histological classification of breast tumors is based on their anatomical origin, and according to the World Health Organization (WHO) in 2012, breast cancers were classified into breast carcinoma and breast sarcoma. Breast carcinomas are breast cancers originating from the breast’s epithelial cell-based components, while breast sarcomas are breast cancers arising from the connective tissues [5]. Breast carcinomas can be in situ (localized in the lobule) or invasive (penetrating neighboring tissues). Based on morphology, invasive carcinomas could be grouped into morphologically identifiable types or no specific type (NST). Histologically, TNBC is a major invasive carcinoma, with only about 10% of morphological types. NST invasive carcinoma is so-called because it does not exhibit enough morphological features. The specific histological types of TNBC are apocrine carcinoma, metaplastic carcinoma, adenoid cystic carcinoma, medullary carcinoma, and invasive lobular carcinoma [5,31].

### 2.2. Molecular Classification

**Figure 1:**
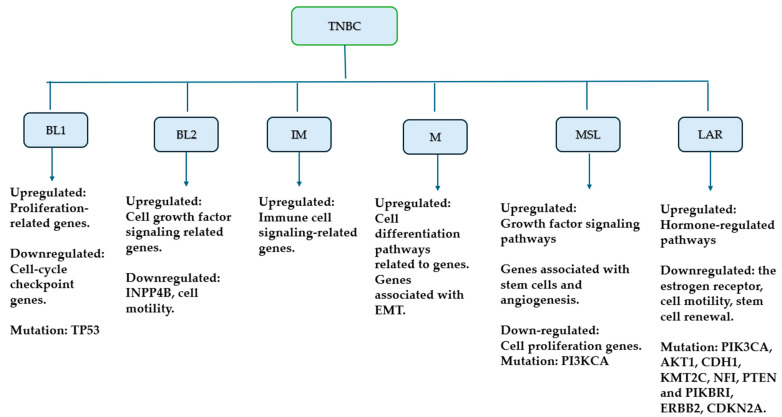
TNBC subtypes and their different gene expression patterns.

Due to molecular heterogenicity [32], TNBC was classified based on gene expression analysis in the spectrum of different groups of TNBCs (Figure 1). The classification and molecular characterization of TNBCs would help to proffer therapeutic strategies centered on personalized treatment [33]. Based on their genetic diversity, multi-omics technology was used by Lehmann and colleagues to classify TNBCs into seven subtypes, namely basal-like 1 (BL1), basal-like 2 (BL2), the immunomodulatory (IM) subtype overexpressing immune signaling genes, luminal androgen receptor (LAR) with AR-activated gene expression, mesenchymal (M) and mesenchymal stem-like (MSL) subtypes characterized by cell motility, angiogenesis-related gene expression, respectively, and an unstable subtype that is unclassified (UNS) [11,33].

Basal-like 1 and basal-like 2: TNBC is a deadly disease with enhanced proliferation and metastatic capability, few treatment choices, and a poor overall result. These tumors are nearly identical to the basal-like molecular subtype and lack expression of known therapeutic targets such as the estrogen receptor, progesterone receptor, and human epidermal growth factor receptor. As a result, these patients have few treatment choices other than cytotoxic chemotherapy, radiation, and surgery [34]. They were most sensitive to cisplatin and showed increased expression levels of proliferation genes, cell cycle checkpoints, and DNA damage response (DDR) genes [34,35]. BL1 showed Bcl-3 gene expression and a high Cathepsin S expression. Bcl-3 increased the drug sensibility of TNBC cells in chemotherapy and also induced G1/S phase arrest through downregulating the protein and mRNA expression of *Skp-2* and *c-MYC*, while upregulating *p27* expression [11]. Also, the BL1 subtypes showed the highest pathological complete response (pCR) from the gene analysis of 300 TNBC patients treated with neoadjuvant chemotherapy compared to the BL2 and LAR subtypes [11].

Immunomodulatory TNBC: They are enriched in immune signaling genes, similar to that of medullary breast cancer, a typical histological type of TNBC that is rare. Immune checkpoint inhibitors can serve as a potential therapeutic method for IM subtypes as they exert their tumorigenesis effect by activating immune suppressive cells or checkpoint molecules [11]. Camrelizumab and nab-paclitaxel have shown promising anti-tumor effects in refractory metastatic immunomodulatory TNBC [36,37].

Mesenchymal and mesenchymal stem-like: These subtypes showed increased genes regulating motility, cell differentiation, and growth factor pathways. The M subtype expresses epithelial–mesenchymal transition (EMT) and cancer stem cells properties. It involves signaling pathways such as extracellular matrix–receptor interaction pathways, Wnt pathways, TGFβ signaling, and breast stem cell biomarkers. M and MSL subtypes have shown sensitivity to the phosphoinositol-3 kinase (PI3K)/mammalian target of the rapamycin (mTOR) inhibitor and the ABL/SRC inhibitor. The co-administration of aminoflavone and histone deacetylase inhibitors can exert an anti-tumor effect on MSL subtypes due to the ability of histone deacetylase inhibitors to sensitize MSL to aminoflavone through improving the reactivity of the aryl hydrocarbon receptor (AhR) [11,34].

Luminal androgen receptors: The luminal androgen receptor (LAR) subtype [38] comprises 15% of TNBC and is enriched for androgen receptor (AR) and AR target genes [39]. LARs are said to have the most significant gene expression changes and are responsive to an AR antagonist, bicalutamide, exhibiting their anti-tumor effect by competitively inhibiting androgen binding to the receptor. LAR patients, compared to other subtypes, showed low proliferation and the poorest pathological complete response (pCR) to the carboplatin regimen. ARs positivity correlate to a greater survival rate, as breast tumors negative of ARs showed a shorter disease-free interval (DFI) and a poor overall survival (OS) when compared to tumors showing a positivity of ARs [11].

## 3. Signaling Pathways in TNBC

### 3.1. Include Targeting PI3K/AKT/mTOR Signaling Pathway

Phosphatidylinositol 3-kinases, a family of intracellular heterodimeric lipid kinases, are among the most abnormally activated pathways in breast cancer that upregulate the expression of anti-apoptosis, while downregulating the expression of pro-apoptosis. The signaling cascade helps to regulate many biological functions like angiogenesis, genomic stability, protein synthesis, cell metabolism, growth, differentiation, proliferation, and survival. The different types of PI3K are categorized into three classes (I, II, and III) based on their biochemical properties, and out of these three classes, class I (PI3Ka, PI3Kb, PI3Kg, and PI3Kd) is considered to include the major PI3K enzymes that are oncogenic [8]. Phosphatidylinositol 4,5-bisphosphate (PIP2) is phosphorylated to phosphatidylinositol 3,4,5-triphosphate (PIP3) through the activation of PI3K, and a plasma membrane increase in PIP3 causes the activation of protein kinase B (AKT) through phosphorylation via phosphoinositide-dependent kinase 1 and 2 (PDK1 and PDK2). The activation of AKT stimulates the mammalian target of rapamycin (mTOR) [40]. Mutations in the phosphatidylinositol 3-kinase (PI3K)/protein kinase B (AKT)/mammalian target of the rapamycin (mTOR) pathway are common in BC (20–40%) and are critical causes of aggressive tumor behavior and therapy resistance. Improving knowledge of the PI3K/AKT/mTOR pathway is necessary [41,42]. The tumor suppressor protein phosphatase and tensin homolog (PTEN) inhibits the expression of PI3K signaling by catalyzing the dephosphorylation of PIP3 to PIP2 [40]. Some studies have evaluated the inhibitors of this pathway for their anticancer effects in TNBC (Table 1).

#### 3.1.1. Buparlisib (BKM120, Norvatis)

**Figure 2:**
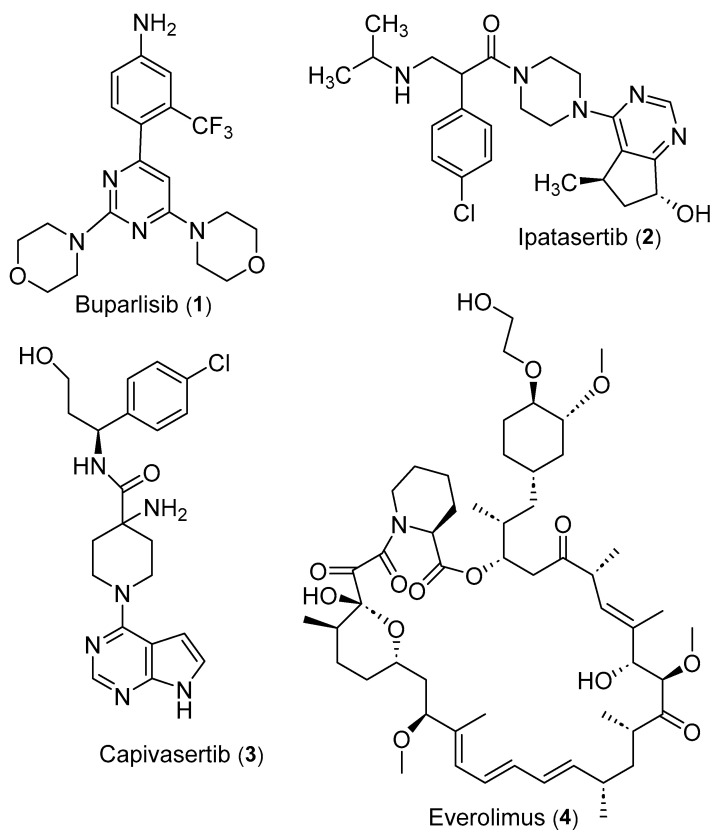
Structures of buparlisib, ipatasertib, capivasertib and everolimus.

Buparlisib (Figure 2) is an orally bioavailable Class I PI3K inhibitor reported to stimulate TNBC tumor regression effectively. The result of a phase 1 dose-escalation study showed 100 mg daily as the maximum tolerated dose. Furthermore, a phase II study was performed to evaluate the clinical activity of buparlisib monotherapy in 50 patients with metastatic TNBC; the result showed a median progression-free survival (PFS) of 1.8 months and a median overall survival (OS) of 11.2 months. The adverse events frequently recorded were fatigue (58%), nausea (34%), hyperglycemia (34%), and anorexia (30%), with 18 patients experiencing depression and anxiety [46]. A standard dose of fulvestrant with or without buparlisib (100 mg/day) was administered to 1147 patients with progressive HR^+^ HER2^−^ MBC after treatment with antiestrogen therapy in a BELLE-2 trial; the buparlisib group showed a median PFS of 6.9 months, as compared to that of the placebo (5 months) [47]. The adaptive phase II/III BELLE-4 study of buparlisib combined with paclitaxel showed no clinical benefits of the co-administration of buparlisib and paclitaxel [48]. Buparlisib induces the inhibition of tumor cells in an ATP-competitive manner by inhibiting class I PIK3, thereby decreasing the production of the phosphatidylinositol (3,4,5)-trisphosphate [49].

#### 3.1.2. Ipatasertib

Ipataserib (Figure 2) is a potent small-molecule kinase inhibitor that shows selectivity for AKT. It competes for ATP and is sensitive to high levels of phosphorylated AKT and mutations in PIK3CA and PTEN, while its resistance is seen in KRAS and BRAF mutations. The use of ipatasertib and chemotherapy was reported to be safe in a phase I trial [44]. The addition of ipatasertib to paclitaxel as a first-line therapy in TNBC in a phase II randomized placebo-controlled LOTUS trial showed an improved median PFS (from 4.9 m to 6.2 m), with a better effect noticed in patients with *PIK3CA/AKT1/PTEN*-altered tumors [50]. However, there was no significant increase in pCR rate upon adding ipatasertib to paclitaxel, as shown in a FAIRLINE trial in early TNBC [51].

#### 3.1.3. Capivasertib (AZD5363)

Capivasertib (Figure 2) is a novel pyrrolopyrimidine-derived small molecule inhibitor that inhibits all forms of AKT. Its sensitivity depends on PI3K/AKT activation and the deletion of PTEN. It inhibits substrate phosphorylation by AKT and decreases the phosphorylation levels of AKT downstream substrates GSK3β and PRAS40 in several cancer cells [52]. In the PAKT trial, the efficacy of adding capivasertib to paclitaxel as a first treatment of TNBC was investigated; the result showed a significant improvement in PFS and OS, with this effect being more evident in patients withPIK3CA/AKT1/PTEN-altered tumors. The median PFS for paclitaxel and capivasertib was 5.9 months, compared to the 4.2 months seen in paclitaxel and placebo. The median OS was 19.1 months for capivasertib and paclitaxel, and 12.6 months for placebo and paclitaxel. In patients with PIK3CA/AKT1/PTEN-altered tumors, the median PFS was 9.3 months for capivasertib and paclitaxel, and 3.7 months for placebo and paclitaxel. Capivasertib was reported to be safe, as the most common grade > 3 adverse events were acceptable–fatigue, rash, infection, neutropenia, and diarrhea [45].

#### 3.1.4. Everolimus (RAD001)

Everolimus (Figure 2), a small-molecule derivative of sirolimus (rapamycin), has been shown to have anti-tumor activities due to its high affinity for the intracellular receptor FKBP12. The binding of everolimus to FKBP12 inhibits mTOR, thereby preventing cell cycle growth, progression, and proliferation [53]. Some metabolic changes were observed in responsive TNBC xenografts treated with everolimus compared to untreated xenografts. Higher levels of glucose, glutamine, and alanine and lower levels of phosphocholine, glycerophosphocholine, and lactate/glucose were noticed in everolimus-treated xenografts. PIK3CA-mutated cells have been reported to show dependence on glucose and glutamine levels for growth. The activation of PI3K increases glucose consumption, further driving more lactate production in tumor cells. The observed glucose increase with decreased lactate levels in everolimus-treated xenografts suggests that the inhibition of mTOR led to a decreased glucose consumption. The decline in phosphocholine was due to the positive effect of everolimus treatment, as phosphocholine (an intermediate in synthesizing the cell membrane phospholipid phosphatidylcholine) increases malignancy [54]. A phase I/II clinical trial, which aimed to assess the effect of adding everolimus to gemcitabine/cisplatin in patients with metastatic TNBC, reported that the addition of everolimus showed no synergistic impact on the patients, as there was no significant difference in pathologic complete response (CR) of patients treated with Gemcitabine/cisplatin plus everolimus (CR = 30.4%) compared to those treated with only gemcitabine and cisplatin (CR = 25.9%). The median PFS and OS for patients treated with gemcitabine/cisplatin plus everolimus were 5.5 months and 19.1 months, respectively, which showed no significant difference for the gemcitabine/cisplatin group (PFS = 5.7 months, OS = 19.1 months) [55]. Conversely, the result from a phase II clinical trial (NCT01127763) showed that the addition of everolimus to carboplatin was efficacious in metastatic TNBC, with dose-limiting hematological toxicity observed with a higher dose of carboplatin (>AUC4) [56].

### 3.2. Targeting Vascular Endothelial Growth Factor (VEGF) Pathway

VEGF is an influential factor in angiogenesis (the formation of new blood cells from old vessels). Solid tumors are said to undergo angiogenesis through secreting pro-angiogenic factors that cause the formation of tumors with a high microvessel density and a poor prognosis because of increased cell growth and metastasis. Angiogenesis facilitates the development of tumors, as tumors depend on blood vessels for nutrients, oxygen, and the excretion of waste products. To this effect, angiogenesis is now a promising strategy for cancer treatment. VEGF is one of the pro-angiogenic factors that enhances vascular permeability, and higher levels of VEGF have been reported in TNBC than in non-TNBC. VEGF drives malignant stem cells, as its upregulation enhances the self-renewal of cancer stem cells after therapy. Its inhibition can stop the supply of oxygen and nutrients necessary for forming new blood cells [11,17]. The VEGF family consists of five isoforms (VEGF-A, VEGF-B, VEGF-C, VEGF-D, and placenta growth factor), with VEGF-A being the most studied isoform. Vascular endothelial growth factor receptor-2 (VEGFR-2) is a tyrosine kinase receptor that regulates VEGF signaling. The binding of VEGF to VEGFR-2 causes the autophosphorylation and dimerization of two VEGFR-2 monomers, leading to the recruitment of VEGF-receptor proteins that activate PI3k through SRC. The activation of PI3k further leads to the induction of anti-apoptotic and cell survival effects due to the activation of AKT [43,57].

#### 3.2.1. Vandetanib

**Figure 3:**
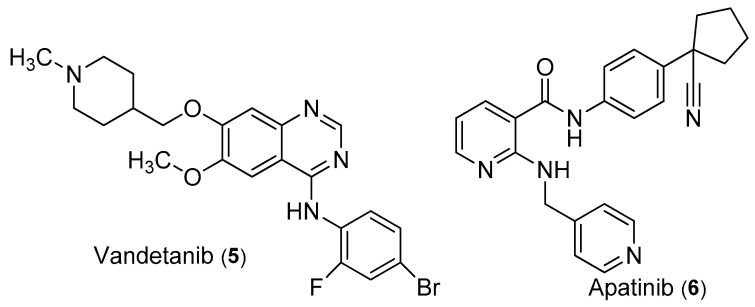
Structures of vandetanib and apatinib.

Vandetanib (Figure 3), a selective inhibitor of VEGFR2, is an orally bioavailable 4-anilinoquinazoline that has been reported to induce anti-tumor activity through downregulating the expression of the VEGFR gene, inhibiting angiogenesis, and inducing TNBC cell necrosis. The phase I combination therapy of vandetanib and metronomic chemotherapy reported the maximum tolerable dose of vandetanib as 200 mg with modest clinical activity. The toxicities recorded were mild, and they included nausea, vomiting, LFT abnormalities, fatigue, and rash [58]. Ling and colleagues also confirmed that vandetanib inhibits the cancer cell growth in the breast by upregulating apoptosis, as the secretion of VEGF was reduced in treated cells [59].

#### 3.2.2. Apatinib (*N*-[4-(1-Cyano-cyclopentyl) Phenyl]-2-(4-pyridlmethyl)amino-3-pyridine Carboxamide)

Apatinib (Figure 3), an oral highly potent tyrosine-kinase inhibitor targeting VEGFR2 was observed to show the synergistic effect on anti-tumor activities of cisplatin on MDA-MB-231 TNBC cells by downregulating the levels of VEGFR2, AKT, and mTOR [60]. Xinchun et al., in a phase II study, evaluated the maximal dose for the efficacy and safety of apatinib monotherapy in heavily pretreated patients with metastatic TNBC in China, and the recommended dose was established as 500 mg/day. The usual occurring toxicities were hand-foot syndrome, proteinuria, hypertension, and increased ALT. The overall response and clinical benefit rates (CBRs) were 10.7% and 25.0%, respectively, for n = 56. The median PFS and overall survival were 3.3 months and 10.6 months, respectively [61].

### 3.3. Targeting Poly (ADP-Ribose) Polymerase (PARP) Pathway

PARP is a polymerase enzyme that regulates gene stability and the repair of single-strand DNA breaks (SSBs) through a base excision repair mechanism. The upregulation of homologous recombination (HR) is used as an alternative means for genetic repair during the inhibition of PARP. Using PARP inhibitors (PARPis) can lead to SSB accumulation and the replication fork’s breaking, but the resulting double-strand DNA breaks (DSBs) can be repaired via HR. However, in breast cancer (BRCA)-mutated BC, PARP inhibiting agents aid in tumor cell deaths through synthetic lethality (where the loss of two genes with similar function leads to cell death, while the loss of one of the genes has little or no lethal effect) in cells expressing HR deficiency (HRD) [34,62]. Two types of BRCA genes exist—BRCA1 and BRCA2. The BRCA1 gene controls cellular pathways involving gene transcription, cell proliferation, and DNA repair response, while the BRCA2 gene controls DNA repair. BRCA genes are responsible for the regulation of HR. Therefore, they act as tumor suppressor genes, as mutations have been observed in tumor cells due to genomic instability, and approximately 80% of BRCA1 and 3–17% of BRCA2-related BC belong to the TNBC subtype [5]. PARP inhibitors (Table 2) show efficacy for the treatment of BRCA-mutated breast cancer and have been evaluated in several clinical studies. PARPi causes cytotoxicity by preventing the release of the PARP genes bound at SSB, forming a PARP–DNA complex. The formation of the PARP–DNA complex precludes the release of PARP genes for further DNA repair activities. Several PARPis have been evaluated, and their potency depends on their ability to trap PARP onto the DNA site. Some PARPis in clinical studies include olaparib, niraparib, veliparib, talazoparib, rucaparib, etc., with veliparib having the least potency compared to others. Talazoparib, which is obtained from a by-product, is the most potent PARP inhibitor and is about 100 times more potent than olaparib (the first clinically evaluated PARPi) and the FDA has approved both for use in the treatment of metastatic cancer with germline BRCA mutations [34,62]. Although PARPis show cytotoxicity to cancerous cells, they have limited use due to the hematologic toxicity imposed on healthy cells. Some clinical studies have shown that the administration of veliparib, which has a lower PARP inhibitory effect with platinum-based chemotherapy, showed lesser hematologic toxicity than the combination of talazoparib or olaparib with platinum-based chemotherapy [62]. Veliparib and niraparib are selective inhibitors of PARP1 and PARP2, as compared to other PARP inhibitors (olaparib and talazoparib), which exhibit an unselectivity to PARP1 inhibitors. Both veliparib and niraparib have demonstrated cellular activity. They engage extensively with the active sites of PARP1 and PARP2, forming unique hydrogen bonds directly and through water mediation. They also exhibit conserved interaction in the nicotinamide binding pocket, a feature shared with most PARP inhibitors. Veliparib and niraparib establish crucial interactions with the α-helical regulatory subdomains (E763 or D766 of PARP1, and E335 of PARP2), needed for their selectivity towards PARP1 and PARP2, since these carboxyl side chains are distinct and not found in other members of the PARP family [63].

#### 3.3.1. Olaparib

**Figure 4:**
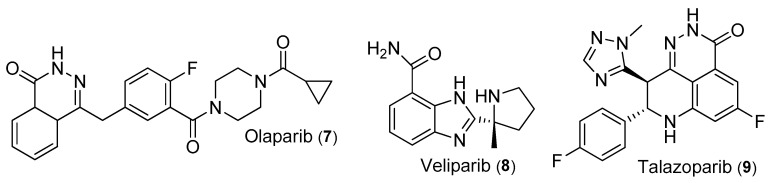
Structures of olaparib, veliparib, and talazoparib.

Olaparib (Figure 4) was the first PARP inhibitor approved in 2018 by the FDA for the treatment of cancer in patients with metastatic breast cancer and a germline BRCA mutation from the result of the OlympiAD trial (a randomized, controlled, open-label, multicenter, international phase 3 trial) [62]. The trial compared olaparib monotherapy to standard chemotherapy (capecitabine, eribulin, or vinorelbine) in patients with BRCA mutation and HER2-negative patients who had received no more than two chemotherapies earlier [66]. Patients had prior anthracycline and taxane chemotherapy, and those that were HER2 positive had received endocrine therapy and had disease progression. Neoadjuvant or adjuvant platinum therapy was permitted if the the time of the last dose administered has passed at least 12 months , and for metastatic disease if no disease progression was observed during treatment. A total of 302 patients were included, and 205 patients were treated with 300 mg olaparib twice daily. At the same time, 97 were assigned to the standard therapy group, where they received either of the three traditional therapies. The results demonstrated that olaparib showed a significantly improved median progression-free survival of 7.0 months compared to the standard therapy group of 4.2 months (hazard ratio, 0.58; 95% confidence interval [CI], 0.43–0.80; *p* < 0.001). The olaparib group showed a better response rate of 59.9% than the 28.8% seen in the standard therapy group. At the same time, the standard group had a grade 3 higher adverse event of 50.5% compared to the 36.6% seen in the olaparib group. Grade 4 and grade 5 adverse events were also higher in standard therapy (12.1% and 1.1%, respectively) than in the olaparib group (3.4% and 0%) [66]. 

The one-year toxicity of the RADIOPARP dose-escalation Phase I trial report evaluated the effect of combining olaparib with radiotherapy for the treatment of TNBC, and the results demonstrated no grade ≥ 3 toxicity, and there was no record of cardiac, pulmonary, and digestive toxicity [67]. Furthermore, Marijo et al. showed that the co-administration of Selinexor (an oral selective inhibitor of nuclear export that exerts its apoptotic effect by inducing the accumulation of tumor suppressor proteins, while blocking the oncogenes responsible for cell proliferation) and olaparib showed a synergistic anti-tumor effect on TNBC, with or without BRCA mutations [68].

#### 3.3.2. Veliparib (ABT-888)

Veliparib (Figure 4) is a potent, orally bioavailable, selective inhibitor of PARP that inhibits poor trapping of the PARP protein onto the DNA site, thereby making it the most effective PARPi for combination with platinum-based chemotherapy, as PARP trapping can lead to myelosuppression [69]. Veliparib has shown minimal toxicity as a monotherapy and in combination with other treatment agents, including carboplatin, cisplatin, paclitaxel, and cyclophosphamide [70,71,72]. The result of a phase II trial for treating metastatic TNBC using cisplatin with or without veliparib demonstrated that adding veliparib to cisplatin significantly improved PFS and OS in BRCA-mutated TNBC. The addition of veliparib improved the PFS from 4.3 months to 5.7 months and the OS from 12.1 months to 13.7 months, respectively, for the BRCA-mutated groups. At the same time, no effect of veliparib was noticed on the non-BRCA mutated group [73]. In the California Cancer Consortium trial (NCT01149083), the efficacy of veliparib monotherapy combined with carboplatin was evaluated for treating BRCA-mutated BC [64]. BRCA-mutated metastatic breast cancer (MBC) patients who had not received prior therapy with either platinum or PARPis were used. In phase II, the response rate and median PFS were 14% at 3.6 months and 36% at 6.6 months in the BRAC1 and BRAC2, respectively. An excellent tolerable toxicity was observed for monotherapy, with a shallow ≥ grade 3 toxicity. An overall response rate (RR) and complete response (CR) were observed in patients treated with upfront veliparib and carboplatin, rather than with veliparib monotherapy. The authors further hypothesized that the upfront administration of veliparib and carboplatin shows a higher synergistic efficacy than the progression treatment of the said combination in patients previously treated with veliparib monotherapy. The tumor during the monotherapy session would have developed other repair mechanisms that would resist the combination therapy’s potential synergy, thereby reducing its efficacy [64]. A randomized, double-masked, placebo-controlled phase 3 trial (BROCADE3) demonstrated that adding veliparib to platinum chemotherapy improved PFS in BRCA-mutated BC patients [69].

#### 3.3.3. Talazoparib

Talazoparib (Figure 4) is a PARPi (poly(ADP-ribose) polymerase inhibitor) that exhibits its anti-tumor activity by firmly trapping PARP onto damaged DNA, thereby causing cell death in BRCA-mutated BC; it has been reported in a two-part, phase I, first-in-human trial to exert a 50% objective response rate (ORR), a PFS of 34.6 weeks, and one complete response in 14 BRCA-mutated BC patients treated with 1.0 mg/day talazoparib [74]. A phase III open-label EMBRACA study compared the efficacy and safety of talazoparib monotherapy to the physician’s choice standard therapy (capecitabine, eribulin, gemcitabine, or vinorelbine), and the patient’s reported outcome indicated that talazoparib monotherapy showed a significantly improved median PFS (8.6 months) than the standard therapy agents (5.6 months), as well as a higher ORR in the talazoparib group (62.6%) than in the standard therapy group (27.2%) [65]. Adverse events grade 3–4 hematologic toxicity, especially anemia, was 55% and 38% in the talazoparib and standard therapy group, respectively [75]. Talazoparib also showed a clinical anti-tumor activity in patients that had been earlier treated with platinum or non-platinum chemotherapy in the phase II ABRAZO clinical trial [76].

### 3.4. Targeting Janus Kinases (JAKs)/Signal Transducer and Activator of Transcription 3 (STAT3) Pathway

The activation of JAK/STAT3 is known to cause cell proliferation, a poor prognosis in BC, and is found to be overly expressed in TNBC, making its inhibition a promising therapeutic target. JAK is a non-receptor tyrosine kinase consisting of four isoforms (JAK1, JAK2, JAK3, and TYK2) [17]. JAKs regulate biological processes like DNA transcription and the expression of proteins by transferring extracellular signals to the nucleus. STAT3 is a downstream effector of different receptor tyrosine kinases (RTKs), and the phosphorylation of STAT3 usually activates the JAK/STAT3 pathway. The binding of cytokines to cytokine receptors can activate JAKs, which further induces the phosphorylation of STAT3. The phosphorylated STAT3 is then translocated inside the nucleus, where the expression of genes related to cancer stem cells [8,77]. Different small molecules have been reported to exert anti-tumor effects by inhibiting the JAK/STAT3 pathway. Examples of JAK/STAT3 inhibitors (Table 3) include Ruxolitinib, Salinomycin, Flubendazole, etc.

#### 3.4.1. Ruxolitinib

**Figure 5:**
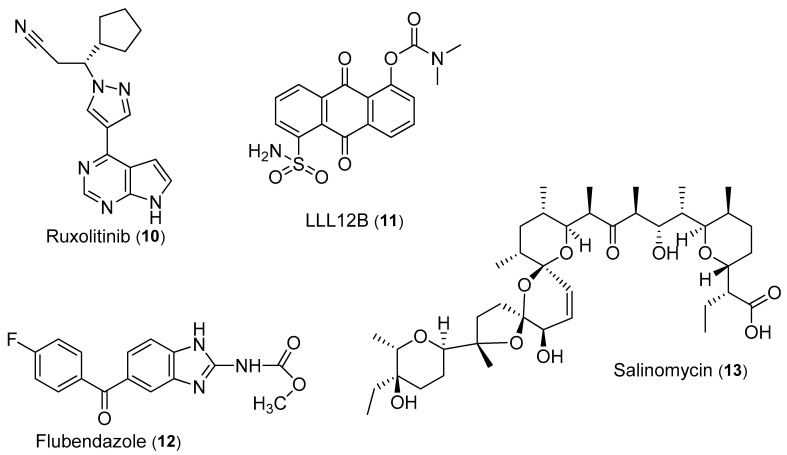
Structures of ruxolitinib, LLL12B, flubendazole, and salinomycin.

Ruxolitinib (Figure 5), an orally bioavailable inhibitor of JAK1 and JAK2, has been approved for use in treating some diseases, including myelofibrosis and polycythemia vera, showing inadequate response or a lower tolerance to hydroxyurea. In 2018, Stover and colleagues, in a non-randomized phase II study, evaluated the efficacy and safety of ruxolitinib (Figure 5) in TNBC patients who were STAT3 positive [79]. They observed that the administration of ruxolitinib monotherapy showed no efficacy, as no objective response was recorded, with a median PFS of 1.2 months. However, ruxolitinib suppressed JAK2-induced genes and reduced the level of STAT3. Based on their findings, they hypothesized that the low anti-tumor efficacy of ruxolitinib might have been due to several factors, including but not limited to its inability to completely inhibit JAK-STAT signaling, cytostatic rather than cytotoxic effects, and intra-tumoral resistance. Hence, ruxolitinib might show a synergistic effect in combination with chemotherapy or other targeted agents [80]. Ruxolitinib demonstrated a synergistic effect in combination therapy with MK-2206 (PI3K/AKT inhibitor) in MDA-MB-231 BC cell lines. The combined therapy decreased metastasis and cell viability, while inducing apoptosis [81].

#### 3.4.2. LLL12B

LLL12B (Figure 5), an orally bioavailable carbamate prodrug of LLL12, has been reported as a novel therapeutic agent for the treatment of TNBC. After the administration of LLL12B, the active drug (LLL12) is released through hydrolysis of the carbamate ester bond [78]. LLL12B was tested in TNBC cells, and the results showed that targeting STAT3 with LLL12B induced apoptosis and suppressed colony formation, migration, and tumor growth in TNBC cells. These findings suggest that LLL12B is a novel therapeutic agent for potential TNBC therapy. The inhibitory effect of LLL12B on STAT3 was evaluated in both in vivo and in vitro studies, and the in vitro result revealed that LLL12B induced apoptosis and inhibited colony formation and cell migration, while suppressing tumor growth in vivo in TNBC cells [78].

#### 3.4.3. FLU

Several preclinical and clinical studies have studied flubendazole (FLU) (Figure 5) potency as an anticancer drug. FLU is an anti-infection drug that is being used to treat intestinal parasites and worms. A study aimed at determining the mechanism of anti-tumor potency of FLU in TNBC reported that the treatment of FLU at 0.1–0.5 µM led to the inhibition of cell growth and G2/M phase cell cycle arrest in TNBC cells. FLU exerted its anti-proliferative effects through the inhibition of tubulin polymerization, leading to a dysfunction in the activation of STAT3 [82].

#### 3.4.4. Salinomycin

The salinomycin (Figure 5) antibiotic was reported to have an anti-tumor effect in MDA-MB-231 TNBC cells through a reduction in the population of CD44^+^/CD24^−^ stem-like cells, while increasing anoikis (a type of cell death caused by detaching the cell from the extracellular matrix); however, its mechanism in TNBC cells is yet to be fully elucidated [80].

### 3.5. Targeting Mitogen-Activated Protein Kinases (MAPK) Pathway

Also known as the RAF/MEK/ERK pathway, MAPK is a signaling pathway consisting of different enzyme-linked serine/threonine protein kinases (RAF, MEK, and ERK) when activated through phosphorylation, regulating cell growth, proliferation, differentiation, and migration, thereby increasing the population of cancer stem cells [8]. RAS, a guanine nucleotide-binding protein, transduces extracellular stimuli into intracellular signals, causing Raf’s phosphorylation, which causes the downstream activation of MEK and ERK. The activated ERK further phosphorylates other pathways, leading to cell differentiation and tumor growth, and the upregulation of Ras is seen in 30% of human cancers [4]. The MAPK pathway is commonly dysregulated in cancer cell therapy, making it a druggable target for the treatment of cancer [17]. Some MAPK inhibitors (Table 4) are being discussed below.

#### 3.5.1. E6201

**Figure 6:**
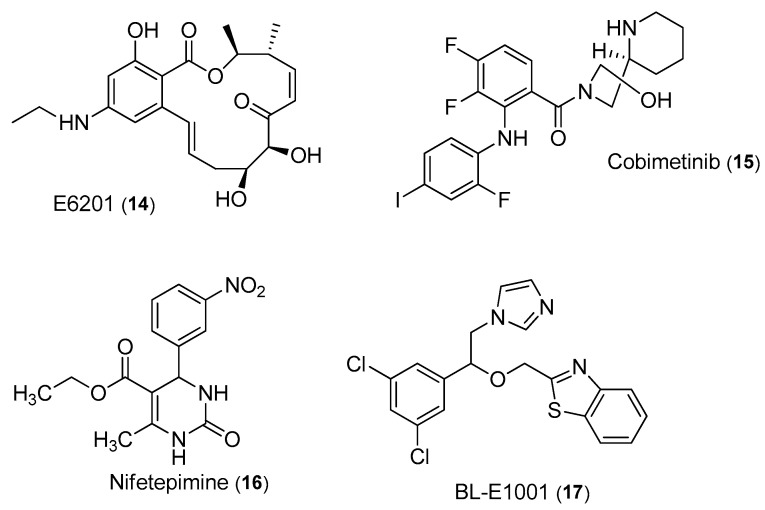
Structures of E6201, cobimetinib, nifetepimine, and BL-EI001.

E6201 (Figure 6), an ATP-competitive dual kinase inhibitor of MEK1, has been reported as a potential treatment agent in MEK-upregulated cancer cells. Dose-dependent growth inhibition and decreased ERK expression in TNBC cell lines after administration of E6201 for 5 days were reported by Jangsoon and colleagues. E6201 induced apoptosis and suppressed tumor cell growth in SUM149 and MDA-MB-231 cell lines [83].

#### 3.5.2. Cobimetinib

Cobimetinib (Figure 6) is an MEK inhibitor that has been reported in a three-cohort phase II COLET (NCT02322814) to ascertain its anti-tumor efficacy in combination with chemotherapy with or without atezolizumab in advanced or metastatic TNBC patients [86]. The upregulation of MAPKs in TNBC causes resistance to standard chemotherapy. The inhibition of this pathway might increase TNBC sensitivity to taxanes and PDL1 (programmed death-ligand 1) inhibitors. Cohort I assessed the co-administration of cobimetinib plus paclitaxel versus placebo plus paclitaxel. Cohorts II and III evaluated the combination of cobimetinib plus atezolizumab plus paclitaxel or nab-paclitaxel. The standard grade ≥3 adverse event was diarrhea. In cohort I, the median PFS for cobimetinib plus paclitaxel and placebo plus paclitaxel was 5.5 months and 3.8 months, respectively. The ORR in cohort I was 38.3% (cobimetinib plus paclitaxel) and 20.9% (placebo and paclitaxel), but was 34.4% for cohort II and 29.0% for cohort III [84,87].

#### 3.5.3. Nifetepimine

Nifetepimine (Figure 6), a dihydropyrimidone derivative (ethyl-4-(3-nitro)–phenyl-6-methyl-2-oxo-1,2,3,4-tetrahydropyrimidine-5 carbo—xylate) [88], caused endoplasmic reticulum (ER) stress-induced apoptosis in TNBC cells by downregulating ERK expression and GRP78 gene transcription both in vivo and in vitro. The upregulation of GRP78 has been associated with tumor growth development and chemotherapy resistance [89].

#### 3.5.4. BL-EI001

BL-EI001 (Figure 6), a small molecule inhibitor, has been reported to induce mitochondrial apoptosis, independent of the Ras/Raf/MEK pathway in MCF-7 breast cancer, by inhibiting ERK phosphorylation [85].

### 3.6. Targeting Epidermal Growth Factor Receptor (EGFR) Pathway

The epidermal growth factor receptor (EGFR) is a transmembrane glycoprotein of the HER family transmembrane receptors involved in different biological processes. There are different types of HER family (ERBB2/HER2, ERBB3/HER3, and ERBB4/HER4), and each consists of a ligand-binding site, transmembrane region, and EGFR membrane tyrosine kinase region. EGFR binds to its ligand (epidermal growth factor, EGF, and transforming growth factor, TGF-α), causing its dimerization and autophosphorylation, and this activates downstream signaling cascades that control cell proliferation, differentiation, migration, angiogenesis, apoptosis, and survival. The abnormal expression of EGFR is related to tumorigenesis in different types of cancer, including TNBC [17,90]. An increased level of EGFR is said to cause poor prognosis in about 80% of TNBC patients, of which ¼ and ¾ are caused by its gene amplification and overexpression, respectively, thereby making it a druggable target for cytotoxic therapy [11]. Some studies have evaluated the inhibitors of this pathway for their anticancer effects in TNBC (Table 5).

#### 3.6.1. Cannabidiol (CBD)

**Figure 7:**
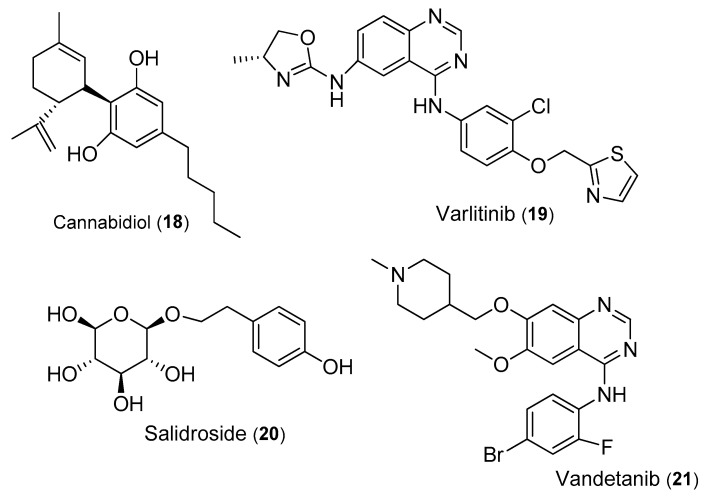
Structures of cannabidiol, varlitinib, salidroside, and vandetanib.

CBD (Figure 7), a non-psychotropic phytocannabinoid, has been reported to show anticancer activities in breast cancer. CBD is a type of cannabinoid (a group of bioactive compounds that bind to specific G-protein coupled receptors) that is obtained from the plant *Cannabis sativa* (a member of the Cannabaceae family, also known as marijuana) [95]. Cannabinoids are classified into three categories—phytocannabinoids (plant-derived), endogenous cannabinoids (from animal and human tissues), and synthetic cannabinoids (made in the laboratory) [96]. CBD induces programmed cell death, decreases ID1 (metastatic) factors, increases ID2 (pro-differentiation) factors, and acts as an inverse agonist for CB2, as well as an antagonist for CB1 and GPR55 receptors, all being G-protein coupled receptors [91]. Although the mechanism of anti-tumor activity of CBD in TNBC cells is yet to be fully elucidated, Elbaz et al. reported that CBD exerts its activity by inhibiting the EGF-induced activation of NF-κβ, migration, ERK, and AKT signaling pathways. The inhibition of NF-κβ activation reduced the expression of CXCR-4 (a receptor used for breast cancer cell migration) and reduced metastasis in BC cells by preventing epithelial–mesenchymal transition (EMT) [91].

Patel and colleagues also reported, in an in vitro study, that the mono-administration of CBD or its combination with extracellular vesicles (EVs) significantly improved the sensitivity of MDA-MB-231 cells to doxorubicin (* *p* < 0.05). Also, the combined administration of CBD, EVs, and doxorubicin decreased the expression of proteins related to inflammation and metastasis, while increasing those related to apoptosis [97].

#### 3.6.2. Varlitinib (ASLAN001)

Varlitinib (Figure 7) is a potent reversible HER inhibitor of the receptor tyrosine. It was reported to significantly induce cell apoptosis and inhibit EGFR, AKT, MEK, and ERK activation in MDA-MB-453 and MDA-MB-468 TNBC cells [92].

#### 3.6.3. Salidroside (*p*-Hydroxyphenethyl-β-*d*-glucoside)

Salidroside (Figure 7, a compound derived from Rhodiola rosea, has been demonstrated to decrease metastasis and invasion in BC through its ability to regulate the STAT3, Jak2, and EGFR pathways through matrix metalloproteinases (MMPs). Salidroside exerted a concentration-dependent cytotoxic effect, as 40 µM salidroside killed 49% of MDA-MB 231 TNBC cells. Salidroside may have no toxic effect on healthy cells, as no cytotoxic effect was noticed on MCF-10A and HUVECs, making them promising BC therapeutics [98]. Also, at a higher dose, salidroside (80 mg/kg) showed a 75.16% tumor inhibitory effect, and this was higher than that of paclitaxel and saline in MCF-7 BC mice [94]. Another study reported the dose-dependent inhibitory effect of salidroside on cell proliferation and colony formation in MCF-7 cells. The IC_50_ value was recorded as 19.48 µM. A significant inhibitory effect was noticed at one µM, while the maximum inhibitory effect was 50 µM [93].

#### 3.6.4. Vandetanib

In an in vivo study, vandetanib (Figure 7) was reported to induce tumor regression in patient-derived xenograft ER-negative BC models, expressing high levels of EGFR or RET (rearranged during transfection) through the inhibition of EGFR phosphorylation. Also, the vandetanib administration reduced tumors but could not induce tumor regression in models not expressing EGFR or RET [99]. Also, vandetanib was reported to show a synergistic anti-apoptotic effect with ixabepilone (a cytotoxic agent) in 231C and TXT cells MDA-MB-231 BC cells showing parental and docetaxel drug resistance. Additionally, the 16.5 nM monotherapy of ixabepilone decreased, as a single agent decreased the levels of live cells from 92.2 ± 0.9% to 50.8 ± 3.5% (*p* < 0.001), while the combination therapy of ixabepilone with vandetanib (1.5 µM) decreased the level of live cells to 27 ± 5% [100].

### 3.7. Targeting Src Pathway

SRC, encoded by the sarcoma gene, is a non-receptor tyrosine kinase and the proto-oncogene *c-Src* product involved in cell survival, angiogenesis, proliferation, and motility. The overexpression of *Src* kinases has been seen in different cancer cells, leading to poor prognosis and metastasis. Other Src inhibitors have been reported to aid in overcoming resistance to chemotherapy in TNBC [101]. We have discussed some of the Src inhibitors (Table 6).

#### 3.7.1. Dasatinib (BMS-354825)

**Figure 8:**
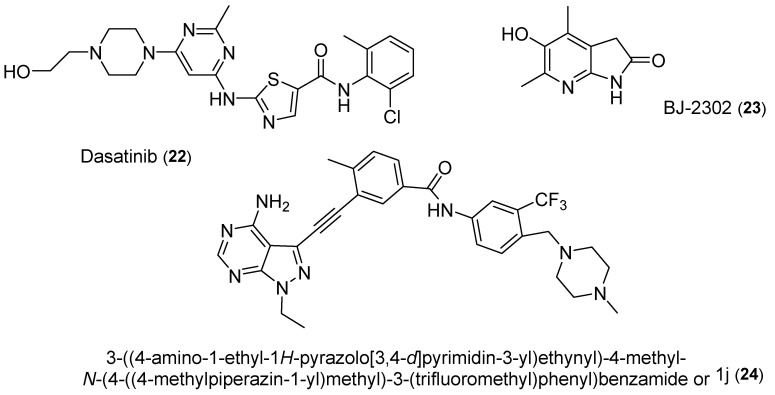
Structure of dasatinib, BJ-2302, and 1j.

Dasatinib (Figure 8), a small molecule inhibitor of Src and abl proteins, has been approved for imatinib refractory chronic myelogenous leukemia and bcr–abl positive acute lymphoblastic leukemia treatment. The anticancer activities of dasatinib in breast cancer were demonstrated in 39 human breast cancer cell lines treated with one μM dasatinib. The human breast cancer cell lines used included luminal and basal breast cancer subtypes and those that went through epithelial-to-mesenchymal transition. A high sensitivity (>60% growth inhibition) was seen in 8 BC cell lines, and moderate sensitivity (40–59% growth inhibition) was seen in 10. At the same time, 21 showed resistance to dasatinib, with basal-type and post-EMT breast cancer cell lines being most sensitive to growth inhibition by dasatinib [105]. Dasatinib was also reported as a suitable therapeutic agent in TNBC cells, showing overexpression of the syndecan-binding protein (SDCBP). SDCBP is a syntenin-1/MDA-9 molecule that regulates metastasis, transmembrane receptors, and neuronal synapse function. Its overexpression upregulates cancer cell progression and the proliferation of MDA-MB-231 TNBC cell lines by increasing the tyrosine phosphorylation of c-src at residue 419 [102]. The result of an open-label phase II study, aimed at determining the efficacy and safety of dasatinib monotherapy in TNBC, reported 70 mg twice daily of dasastinib as its tolerable dose. Of the 44 patients, 2 had confirmed partial responses that lasted up to 14 and 58 weeks. In total, 11 patients had a stable disease, and the median PFS was 8.3 weeks. Grade 3 adverse events were seen in 5% of the patients, with fatigue, diarrhea, pleural effusion, and dyspnea being the most frequent, while grade 4 adverse events were not noticed [106]. In addition to the downregulation of *Src*, dasatinib was shown to exhibit its effect on BC cell lines through decreasing the phosphorylation of EGFR, as dasatinib was able to induce apoptosis in breast cancer cells (MDA-MB-468, SKBR3, MDA-MB-453, and MDA-MB-231) overexpressing EGFR, HER-2, and HER-3. Dasatinib induced apoptosis by activating caspase-9 and -8 and arresting the G_0_/G_1_ cell cycle [107]. Furthermore, dasatinib was reported to show a synergistic effect with paclitaxel in overcoming chemoresistance in BC stem cells. Dasatinib inhibited paclitaxel-induced breast cancer stem cell (BCSC) and deactivated Src in TNBC cells, showing parental and paclitaxel resistance. Dasatinib enhanced the sensitivity of the cancer cell lines to paclitaxel by improving the epithelial differentiation of the mesenchymal cells responsible for pac-resistance [108]. Out of seven TNBC cell lines, six cell lines treated with dasatinib plus cisplatin plus cetuximab (a monoclonal antibody) revealed that the addition of dasatinib exhibited a synergistic apoptotic effect when compared to the two TNBC cell lines with only cisplatin and cetuximab combination. It was demonstrated that adding dasatinib helped improve the sensitivity of EGFR to cetuximab inhibition [27].

#### 3.7.2. BJ-2302

BJ-2302 (Figure 8), a novel 7-azaindolin-2-one derivative, suppressed cell invasion and metastasis in MDA-MB-231 cells by strongly inhibiting Src (IC_90_: 3.23 µM), leading to a decreased expression of lysosomal enzyme cathepsin S (CTSS) and gelatinase matrix metalloprotease (MMP)-9, which are responsible for poor prognosis, reduced survival, and triple-negativity in TNBC cells [109].

#### 3.7.3. Compound 1j (**24**)

1j (Figure 8), a multikinase inhibitor, has been reported among other 3-(phenylethynyl)-1*H*-pyrazolo [3,4-d] pyrimidine-4-amine derivatives to show the highest inhibition of Src kinase and reduced cell viability in MDA-MB-231 TNBC cell lines. Also, 1j showed an inhibitory effect on the MAPK signaling pathway (IC_50_ = 0.0009 μM) and MAPK signaling protein kinases B-RAF and C-RAF. In an MDA-MB-231 xenograft mouse model, the administration of 1j at a daily dose of 30 mg/kg for 18 days, completely decreased tumor size (IC_50_ = 0.0009 μM). The tumor inhibition rate was over 100%, with little or no toxicity noticed [110].

### 3.8. Targeting the E-Cadherin Expression

E-cadherin (CDH1) is a calcium-dependent protein, encoded by the *CDH1* gene in humans that facilitates cell–cell adhesion through homophilic interactions. Its inactivation is a hallmark of the invasive breast cancer phenotype. Some correlation has been seen between reduced *E-cadherin* expression and distant metastases and patient outcomes, but its expression in TNBC is not well understood [111]. It was shown that inhibiting the tyrosine kinase ROS1 is synthetically lethal with E-cadherin deficiency. A study revealed a significant reduction in *CDH1* expression, which was frequently observed in TNBC. TNBC patients with defective E-cadherin expression showed a worse prognosis compared to those with a positive E-cadherin expression. In E-cadherin-negative cells, ROS1 inhibitors caused mitotic and multinucleation abnormalities, which are linked to defects in cytokinesis and irregular p120 catenin phosphorylation and localization. In animal models of E-cadherin-deficient breast cancer, ROS1 inhibitors showed significant effects against tumors [111,112].

### 3.9. Combination Therapy with Small Molecules in TNBC

ADCs are being explored as a promising treatment approach for TNBC. They combine the targeting precision of monoclonal antibodies with the potent cytotoxic effects of chemotherapeutic drugs, aiming to provide a more focused and efficient treatment option for TNBC. Sacituzumab govitecan (Trodelvy) is an ADC that combines the antibody sacituzumab with the small molecule SN-38 (a potent topoisomerase inhibitor) and has been approved for the treatment of TNBC, as it specifically targets tumor-associated calcium signal transducer 2 (TROP-2), ensuring the selective delivery of SN-38. Multiple therapeutic approaches including a combination of small molecules with ADC and immune checkpoint inhibitors are being tested to reduce the drug resistance and dose-dependent toxicity that are seen in the use of small molecule inhibitors. The primary outcome of a phase 1b/2 clinical trial (NCT04039230) showed a dose-limiting toxicity (DLT) for the combined therapy of Trodelvy and talazoparib in TNBC [113].

## 4. Challenges and Prospects

Despite the increase in the use of small molecules as a targeted treatment for TNBC, their usage still has some drawbacks. As with most targeted therapies, small molecules do experience low efficiency and resistance to cancer cell drug-resistant mutations. These anti-cancer drug resistances come in several ways, including cancer stem cells [21], gene mutations, aberrant regulation of apoptosis, autophagy, drug–target alteration, drug inactivation, epigenetics, drug efflux, DNA damage repair, cell death inhibition, epithelial–mesenchymal transition, and the upregulation of other genes [103,104]. Several approaches are being taken to reduce these drawbacks, including combinational therapy (antibody–drug conjugates or ADCs, immunotherapy, and chemotherapy) and targeting cancer stem cells [114]. The results from various research in combination therapy have shown promising activity in treating TNBC, thereby demanding more attention to this area. In a KEYNOTE-162 trial, niraparib (a PARP inhibitor) in combination with pembrolizumab (an immune checkpoint inhibitor) showed an ORR and DCR of 21% and 49%, respectively, in 55 TNBC patients, which was significantly better than a single use of the immune checkpoint inhibitor [115]. Also, mTOR inhibitor (everolimus) has synergistically improved the cytotoxic effect of taxane, a chemotherapeutic agent [116].

## 5. Conclusions

This study reviewed different signaling pathways that are aberrantly activated in TNBC and discussed several small-molecule drugs that target them, including their mechanism of action, limitations, and ways to improve their cytotoxicity level through combination therapy. As discussed herein, many small molecules have demonstrated better potency and selectivity. It is well-known that the ultimate goal of any drug discovery research is to increase the potency with reduced toxicity (side effects). The observed anticancer activity of small molecule inhibitors is believed to be due to their tailored delivery and various molecular mechanisms of action, which include altering the metabolism, producing reactive oxygen species (ROS), and mainly blocking key enzymes that contribute to cell proliferation, angiogenesis, and metastasis in TNBC. With further research, either standalone or by a combination of small molecules with antibody–drug conjugates, immunotherapy and/or chemotherapy might be a promising therapeutic strategy to help combat the drug resistance of TNBC in years to come. In a nutshell, some of the molecules discussed in this article have shown promise in becoming commercial drugs in the future.

## Figures and Tables

**Table 1:** Examples of PI3K/AKT/mTOR/ small molecule inhibitors.

S/N	Small Molecule	Preclinical and Clinical Results	Clinical Stage	Reference
**1**	Buparlisib	p110α (IC_50_; 52 nM), p110β (IC_50_; 166 nM), p110δ (IC_50_; 116 nM), p110γ (IC_50_; 262 nM)	NCT01576666 (phase 1)	[43]
**2**	Ipatasertib,	ORR = 33%	Phase I	[44]
**3**	Capivasertib	Median PFS = 5.9 months OS = 19.1 months	PAKT trial	[45]
**4**	Everolimus	(IC_50_ = 5−6 nM)	NCT01931163 (phase 2), NCT02616848 (phase 1), NCT02456857 (phase 2), NCT02120469 (phase 1), NCT02890069 (phase 1)	[11]

**Table 2:** Examples of PARP inhibitors.

S/N	Small Molecule	Preclinical and Clinical Results	Clinical Stage	Reference
**7**	Olaparib	IC_50_ = 5 nM	Phase I, II	[11]
**8**	Veliparib	PFS = 5.7 months OS = 13.7 months	Phase II	[64]
**9**	Talazoparib	PFS = 8.6 months ORR = 62.6%	EMBRACA Phase III	[65]

**Table 3:** Chemical properties of JAK/STAT3 inhibitors.

S/N	Small Molecule	Pre-Clinical and Clinical Results	Clinical Stage	Reference
**10**	Ruxolitinib	IC_50_ = 3 μM	NCT01562873 (phase 1)	[11]
**11**	LLL12B	Decreased CD44^+^/CD24^−^ Stem-Like Population		[78]
**12**	Flubendazole	MDA-MB-231 (IC_50_ = 0.25 μM), Hs578T (IC_50_ = 0.125 μM), BT-549 (IC_50_ = 0.125 μM)		[11]
**13**	Salinomycin	MDA-MB-231 (IC_50_ = 0.5−10 μM)		[11]

**Table 4:** Examples of MAPK inhibitors.

S/N	Small Molecules	Preclinical and Clinical Results	Clinical Stage	Reference
**14**	E6201	MDA-MB-231 (IC_50_ = 0.25 μM), SUM149 (IC_50_ = 0.21 μM), SUM159 (IC_50_ = 2.36 μM)	None	[83]
**15**	Cobimetinib	IC_50_ = 4.2 nM	None	[84]
**16**	Nifetepimine	IC_50_ = 50 μM	None	[80]
**17**	BL-EI001	IC_50_ = 5 μM	None	[85]

**Table 5:** Examples of EGFR pathway inhibitors.

S/N	Small Molecule	Preclinical and Clinical Results	Clinical Stage	Reference
**18**	Cannabidiol	IC_50_ = 6 μM		[91]
**19**	Varlitinib	MDA-MB-231 (IC_50_ = 0–10 μM)	NCT02338245 (phase 2)	[92]
**20**	Salidroside	(IC_50_ = 40 μM)		[93]
**21**	Vandetanib	(IC_50_ = 40 nM)		[94]

**Table 6:** Examples of SRC inhibitors.

S/N	Small Molecule	Preclinical and Clinical Results	Clinical Stage	Reference
**22**	Dasatinib	IC_50_ = 0.8 nM	NCT02720185 (phase 2)	[102]
**23**	BJ-2302	IC_90_ = 3.23 μM		[103]
**24**	1j	IC_50_ = 0.0009 μM		[104]

## Data Availability

All data are available online. No unpublished data have been used in this paper.

## Abbreviations

ADC, antibody drug conjugate; AhR, aryl hydrocarbon receptor; AKT, activation of protein kinase B; AR, androgen receptor; BC, breast cancer; BL1, basal-like 1; BL2, basal-like 2; BLBC, basal-like breast cancer; BRCA, breast cancer; c-Myc, cellular Myc; CBR, clinical benefit rate; CI, confidence interval; DLT, dose-limiting toxicity; DCIS, ductal carcinoma in situ; DDR, DNA damage response; DFI, disease-free interval; EGFR, targeting epidermal growth factor receptor; EMT, epithelial–mesenchymal transition; ER, estrogen receptor; GSK3β, Glycogen synthase kinase-3 beta; HER2^−^, human epidermal growth factor receptor-2-negative; HER2, human epidermal growth factor receptor 2; HR^+^, hormone receptor–positive; HRD, homologous recombination deficiency; HRP, homologous recombination proficiency; IM, immunomodulatory; JAKs, Janus Kinases; LAR, luminal androgen receptor; M, mesenchymal; MAPK, targeting mitogen-activated protein kinases; MBC, metastatic breast cancer; MITs, morphologically identifiable types; mRNA, messenger RNA; MSL, mesenchymal stem-like; mTOR, mammalian target of rapamycin; NF-κβ, Nuclear Factor-κβ NST, no particular type; OS, overall survival; PARP, targeting Poly(ADP-ribose) polymerase; pCR, pathological complete response; PDK1 and PDK2, phosphoinositide-dependent kinase 1 and 2; PFS, progression-free survival; PI3K, phosphoinositol-3 kinase; PIK3CA, phosphatidylinositol-4,5-bisphosphate 3-kinase; PIK3CA, phosphatidylinositol-4,5-bisphosphate 3-kinase catalytic subunit alpha; PIP2, Phosphatidylinositol 4,5- bisphosphate; PIP3, phosphatidylinositol 3,4,5-triphosphate; PR, progesterone receptor; PRAS40, proline-rich Akt substrate of 40 kDa; PTEN, phosphatase and tensin homolog; RTKs, receptor tyrosine kinases; STAT3, signal transducer and activator of transcription 3; TGFβ, transforming growth factor-beta; TNBC, triple-negative breast cancer; UNS, unstable subtypes; VEGF, vascular endothelial growth factor; Wnt, Wingless-related integration site.
